# Investigating the Physiological Mechanisms between Resistance Training and Pain Relief in the Cancer Population: A Literature Review

**DOI:** 10.4236/jct.2023.142008

**Published:** 2023-02-28

**Authors:** Yvonne Jiang, Peter C. Angeletti, Amy J. Hoffman

**Affiliations:** 1 College of Arts and Sciences, University of Nebraska-Lincoln, Lincoln, NE, USA; 2 Nebraska Center for Virology, University of Nebraska-Lincoln, Lincoln NE, USA; 3 College of Nursing, University of Nebraska Medical Center, Omaha, Nebraska, USA

**Keywords:** Lung Cancer, Breast Cancer, Prostate Cancer, Exercise, Physical Activity, Cancer, Pain, Symptoms, Pain Relief, Resistance Training, Pain Relief, Biochemical Pathway

## Abstract

This literature review examines the mechanisms of how exercise, specifically in the form of resistance training, may lead to pain relief in the cancer population. Primary data from three different cancer populations: breast, prostate, and lung, will be examined. A number of experimental studies have been conducted to confirm the effectiveness of resistance training on pain relief as well as the biochemical pathways that relate to this process. In this review, we will examine 5 randomized controlled trials. For the purposes of this review, pain is defined as physical suffering or discomfort associated with illness. Pain is the body’s natural signal, bringing attention to damage that has been sustained by tissues. However, chronic pain is common in the cancer population, and often serves no good purpose but instead will negatively impact both physical and mental health. The three types of pain: nociceptive, neuropathic, and inflammatory pathways have been investigated, and the knowledge of pain mechanisms allows for the understanding of how it is associated with pain. The purpose of this exploratory literature review is to give insight on how to maximize pain-relieving effects of resistance training. Research has indicated that resistance training modulates pain pathways by upregulating the release of pain-relieving substances including beta-endorphins, anti-inflammatory cytokines, and endocannabinoids. Understanding of the benefits of resistance training may be useful in relieving cancer pain, and reproducing effects of pain-relieving strategies while minimizing the symptoms related to cancer and its treatment.

## 1. Introduction

Over 70% of individuals with cancer experience pain, and it is often reported as one of the most debilitating symptoms [1]. Pain may be directly derived from cancer symptoms or a result of the negative effects associated with treatments such as chemotherapy and radiation. Multiple studies [2] [3] [4] conclude that resistance training is an effective way to manage pain in the cancer population. Research has been conducted on many common types of cancer, and this review will examine impacts of resistance training in the breast, lung, and prostate cancer populations. These three types of cancers have relatively high survival rates, with prostate cancer at >99%, lung cancer at 64%, and breast cancer at 90% 5-year survival rate when the disease is detected while it is localized [5]. Higher survival rates indicate that participants in these studies are likely undergoing treatment with curative intent rather than palliative care. Examination of these three different populations will allow for analysis of the effect biological sex and hormones in results of these interventions as the breast cancer population is predominantly female, the prostate cancer population is predominantly male, and the risk for lung cancer is approximately equal in males and females.

Pain can be categorized into three different types: nociceptive, neuropathic, and inflammatory. Although the three types of pain are related, the unique causes and mechanisms of each type must be understood in order to maximize the effectiveness of pain-relieving treatments. Nociceptive pain is caused by damage to body tissues where sensory neurons relay signals to the thalamus via the spinal cord. This type of pain is experienced as a sharp, aching, or throbbing sensation. Neuropathic pain is caused by direct damage to nerves. This is typically described as a shooting, stabbing, or burning sensation. Cancer drugs such as chemotherapy can often induce neuropathic pain due to the damage to nerve endings and healthy tissue. Inflammatory pain is an immune response mediated by pro-inflammatory cytokines (signaling proteins secreted by the immune system), and causes redness, heat, and throbbing at the affected site [6].

Pain will be the major symptom of interest in this review. About 38% of individuals with cancer experience moderate to severe pain chronically. Pain can be caused by tumor growth which compresses surrounding nerves, bones or tissues. In addition, if cancer metastasizes to surrounding organs, it can lead to blockages of the GI tract, lymph nodes, or surrounding blood vessels [7].

Resistance training is able to regulate the release of various neurotransmitters and signaling proteins involved in pain pathways. For instance, the release of beta-endorphins, an endogenous opioid produced by the pituitary gland, is upregulated (increased flux/expression) during resistance training. This alters pain signaling pathways by inhibiting the release of neurotransmitters such as substance P, a neurotransmitter that modulates pain sensation and the inflammatory response, and prevents signals from reaching the thalamus which is the relay station between the body’s sensory system and the cortex. In addition, resistance training causes muscles to release anti-inflammatory cytokines (such as IL-6, IL-1RA and IL-10 that have antagonistic effects on inflammation). The release of endocannabinoids (endogenous neurotransmitters that bind to tetrahydrocannabinol (THC) receptors throughout the nervous system) is also upregulated during exercise as they interact with pain pathways by binding to cannabinoid receptor type 1 and 2 (CB1 and CB2) receptors. This inhibits the production of excitatory neurotransmitters and pro-inflammatory cytokines in various pain pathways [8].

The goal of this literature review is to confirm the pain-relieving effects of resistance training in the cancer population, and determine the mechanisms that allow this to occur so that pain relieving effects of various drugs can be reproduced naturally. This knowledge will potentially improve the overall quality of life and survivorship of the cancer population as exercise has been associated with reduced pain, improved quality of life, and decreased mortality. In this review, we begin by analyzing primary studies done to investigate the effects of resistance training in the breast cancer, lung cancer, and prostate cancer population. Then, we will discuss the three types of pain (nociceptive, neuropathic, and inflammatory) pathways. After we establish a basis of understanding relating to pain processes and pain perception, we will proceed to examine the mechanisms that lead to pain relief (Beta-endorphins, endocannabinoids, cytokines) and how these mechanisms are affected by exercise.

## 2. Methods

Literature searches were conducted using PubMed and Google Scholar. The inclusion criteria consisted of primary sources and meta-analyses of randomized controlled trials that included participants with cancer or cancer survivors that were undergoing treatment for curative intent or have undergone treatment in the past. Intervention groups were assigned physical exercise in the form of either resistance or aerobic training, and studies included a control group receiving usual care to serve as a baseline of comparison. The search strategy was conducted using the MeSH terms “resistance training,” “cancer,” “tumor,” “pain,” “pain relief,” “resistance training AND cancer,” “resistance training AND pain relief,” “tumor AND pain” “cancer AND pain”. Quantitative data was analyzed for significance using a 95% confidence interval. 5 articles met the inclusion criteria (Table 1).

## 3. Theoretical Framework

The Theory of Symptom Self-management (TSSM) guided this literature review. The TSSM emphasizes the enhancement of perceived-self-efficacy (PSE) in patients and the analysis of interactions between symptoms. In order to maximize pain relief, there should be a focus on enhancing PSE to increase symptom self-management. In addition, interventions will likely achieve more optimal results when taking into account physiological, psychological, and contextual patient characteristics rather than relying on quantitative data alone. Exercise treatments should be monitored to ensure that they are administered at a reasonable intensity, will not worsen other symptoms, and are sustainable in the long-term [9].

## 4. Resistance Training and Pain Relief in Cancer Populations

### 4.1 Breast Cancer

Second to skin cancer, breast cancer is the most common form of cancer in females in the United States which comprises approximately 30% of female cancer diagnoses each year. It is estimated that 51,400 new cases are diagnosed, and about 43,250 women die from breast cancer each year. According to the results of a 2017 cross-sectional study, 71.1% of individuals in the breast cancer population experience pain at a high enough intensity that it significantly decreases overall quality of life. These results were measured by the Global Health Self-assessment, Research and Treatment for Cancer Quality of Life Questionnaire-Core 30, and the Breast Cancer-specific 23 and Short McGill Pain Questionnaire [10]. A 2018 randomized controlled clinical trial was designed to investigate the effects of exercise in the form of both resistance training and aerobic training on participants’ pressure pain threshold (PPT): the minimum amount of force required for a stimulus to induce pain. PPT was measured on the middle trapezius and the gluteus muscles with an electronic algometer. The sample consisted of 240 women 18 – 70 years, breast cancer stages I-III, scheduled to undergo chemotherapy. There were three groups in the study: a control group receiving usual care (n = 60), an experimental group performing moderate intensity aerobic training with high intensity interval training (AT-HIIT) (n = 72), and an experimental group performing resistance training with high intensity interval training (RT-HIIT) (n = 74). Participants were assigned to 16 weeks of RT-HIIT twice per week at a rehabilitation center. Resistance exercises were assigned as 2 – 3 sets of 8 – 12 reps at 80% of 1RM, and progressive overload was imposed (weight was increased incrementally) when participants could perform more than 12 reps. The pressure-pain threshold for each of the 3 groups were measured before and after the intervention with no significant differences found at baseline. The control group receiving usual care had significant reductions in PPT post-intervention. Both the AT-HIIT and RT-HIIT groups had significantly higher PPTs than the control (p < 0.05), but only the RT-HIIT group showed an increase in PPT post-treatment. These results indicated that combining resistance training and high-intensity interval training (RT-HIIT) provides significant benefits in reducing pain hypersensitivity (symptom associated with chemotherapy), as there is a significant, positive correlation between muscle strength and pain sensitivity [3].

Many similar studies have been done to investigate the effects of resistance training on cancer-related pain. For example, the results of a 2019 blinded randomized controlled trial examined the effects of both resistance training and the use of a compression garment on breast-cancer related lymphedema (localized swelling caused by lymphatic blockage). The sample consisted of 60 women ages 18 years and older with breast cancer related to lymphedema symptoms. There were two groups receiving treatment in the study: resistance training alone and resistance training with a shoulder compression garment. Measurements of pain and other lymphedema symptoms were taken at 8 and 12 weeks. Results of statistical analysis indicated significant reduction in lymphedema volume (p < 0.01), increase in shoulder range of motion (ROM) (p < 0.05), and decrease in severity of pain (p < 0.05) at week 8 and week 12 in both groups with no significant differences between groups, indicating that resistance training has beneficial effects on inflammation regardless of whether or not compression is used. Lymphedema results when lymph vessels are blocked or damaged. The use of the compression garment was intended to improve lymphatic drainage and blood circulation. The researchers found that exercise activates the sympathetic nervous system which innervates lymphatic vessels, reducing obstruction and decreasing tension in surrounding tissues. Specifically, resistance training increases protein reabsorption (measured with the micro puncture technique in the kidney), further improving lymphatic flow and decreasing edema [1].

### 4.2 Lung Cancer

Lung cancer is the second most common form of cancer in both men and women. There are about 236,740 new cases each year and about 130,180 deaths per year from lung cancer. The most common form of lung cancer is non-small cell lung cancer (NSCLC) which comprises 84% of cases, and 13% of cases are categorized as small cell lung cancer (SCLC). The results of a 2003 meta-analysis indicate that pain is experienced by 27% of outpatients and 76% of palliative care patients in the lung cancer population. The effects of resistance training in both the preoperative and postoperative stages have been examined on this population as well. Nociceptive pain is most common, although patients have reported neuropathic pain in 30% of cases [11] [12]. In addition to pain, one of the major concerns of lung resection is decrease in physical function which can interfere with ability to perform daily tasks [2]. Implemented an exercise regimen that included a combination of aerobic and resistance training to observe effects on a sample (n = 40) of patients with early stages (I-II) of non-small cell lung cancer (NSCLC). Data was collected before surgery and at 1 and 6 months after surgery with curative intent. The sample consisted of an intervention group (IG) and a control group (CG). The IG participated in 14 exercise sessions in the preoperative period and 39 sessions in the postoperative period. Preoperative sessions included 2 – 3 outpatient sessions per week for 2 – 3 weeks beginning within 30 days from referral, and postoperative sessions included two outpatient sessions per week and 3 home-based sessions per week. Results measured by a 6-minute walk distance test with the IG having significantly higher exercise tolerance at 6 months after surgery (p < 0.001) and significantly lower exercise impairment 1 month after surgery (p < 0.025) [13].

In addition to this, combining resistance training and aerobic exercise is effective in reducing bodily pain after lung cancer surgery. A 2013 randomized, assessor-blinded, controlled trial with a sample of 78 participants post-lung cancer surgery consisted of a CG (n = 37) and an IG (n = 41). Participants in the IG completed sessions once per week for 10 weeks, and sessions included a combination of aerobic exercises, resistance training, and dyspnea management. Aerobic exercise was performed at 60% - 80% of work capacity. The supervised exercise program included a 15-minute warm up, 10 – 20 minutes of aerobic training followed by 15 minutes of resistance exercises targeting the arms, trunk, and legs, and ended with a 10-minute cool-down. Participants performed 1 – 2 sets of 6 – 10 reps of each exercise at a rating of perceived exertion (RPE) of 6. There were no significant differences in lung function between the CG and the IG at 4 or 12 months. However, results indicated there were significant improvements in bodily pain (p < 0.01) in the IG compared to the CG at 4 months post-surgery. Improvements were associated with increased physical functioning and were described as moderate in intensity. The primary outcome was increased quality of life after 4 months of the intervention. Lung function was assessed with the six-minute walk test and spirometry. Bodily pain was measured using the Short-form Health Survey questionnaire (SF-36) [14].

### 4.3 Prostate Cancer

Prostate cancer is the second most common form of cancer in men with about 268,490 new cases per year and about 34,500 deaths per year. Despite the use of pain-management medications, such as opioids including tramadol and morphine, about 40% of individuals with prostate cancer experience pain on a daily basis. Androgen deprivation therapy (ADT) is commonly used to treat various stages of prostate cancer. Amongst other unwanted side effects, ADT is linked to a 23% increased risk of rheumatoid arthritis (RA): an autoimmune condition causing pain and inflammation in the joints. Testosterone has many immunosuppressive roles throughout the body, therefore inhibition of this androgen stimulates uncontrolled, autoreactive T-cell production in the thymus, leading to autoimmune responses [15]. Resistance training stimulates anti-inflammatory mechanisms such as the interleukin-6 (IL-6) pathway which relieves joint pain and inflammation in those who suffer from RA [16] [17]. In the long-term resistance training builds muscle mass which helps regulate testosterone levels. This may lower the risk of RA in patients undergoing ADT.

A 2007 study was done to investigate the effects of exercise in preserving muscle mass in patients with prostate cancer undergoing ADT. The randomized controlled trial included 58 participants, and the IG (n = 28) participated in a 16-week, high-load strength training program while the CG (n = 30) proceeded with usual care. Sessions took place 3 times per week with 2 – 3 sets of 9 exercises targeting upper and lower extremities at various prescribed intensities. Lean body mass (LBM) was measured by dual x-ray absorptiometry (DXA). There were no differences between groups at baseline, and the IG had significantly improved LMB (p < 0.01) compared to the CG in both upper and lower extremities at week 16, indicating that resistance training is able to counteract loss of muscle mass which is a common side effect of ADT [18] (Table 2).

## 5. Types of Pain

In order for pain to be experienced, a sensory neuron must be stimulated and the signal must be relayed to the somatosensory cortex via the spinal cord. The somatosensory cortex is the region of the brain responsible for processing sensory information, in other words, the body feels pain when the signal reaches this area [6]. Neurons are activated by neurotransmitters which lead to action potentials (nerve impulses). During a nerve impulse, there is an influx of sodium ions (Na^+^) (depolarization), followed by an efflux of potassium ions (K^+^) (hyperpolarization). Sensory neurons are activated when a stimulus is strong enough to reach a “threshold” which will generate a nerve impulse to be relayed to the brain [6]. There are three types of pain: nociceptive, neuropathic, and inflammatory.

### 5.1 Nociceptive Pain

Nerve cell endings that are responsible for detecting pain are called nociceptors. Nociceptors can detect chemical, mechanical, or thermal stimuli that are potentially dangerous, and are located in the skin, joints, muscles, bones, and organs.

**Figure 1.**
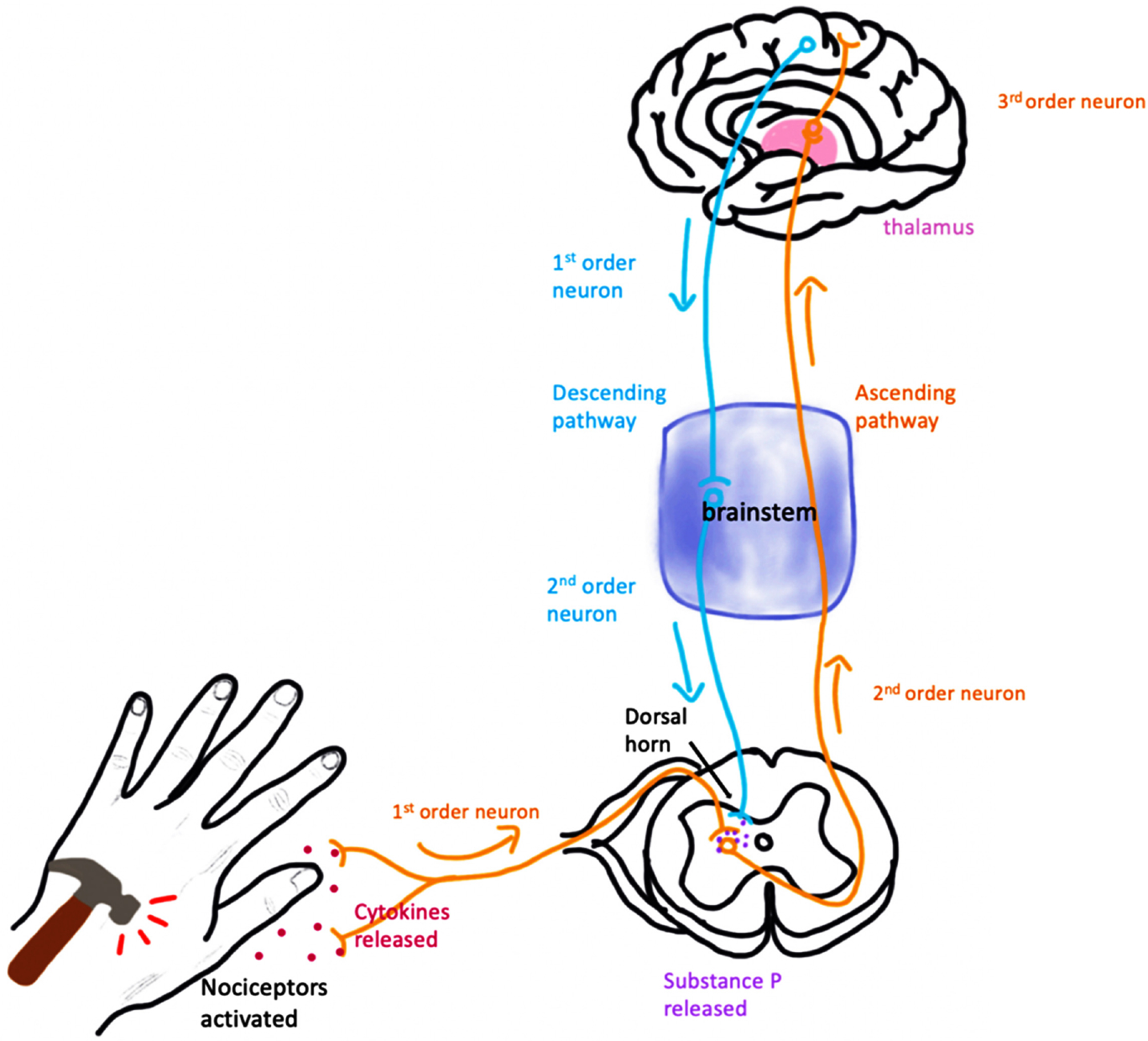
Pain stimulates nociceptors and cytokines are released into the bloodstream to activate the 1st order neuron in the ascending pathway which synapses with the 2nd order neuron at the dorsal horn of the spinal cord. The 2nd order neuron synapses with the 3rd order neuron at the thalamus. The signal is then relayed to the somatosensory cortex and the descending pathway is activated to release substance P at the dorsal horn resulting in the sensation of pain.

There are two routes where pain signals are relayed: the ascending pathway and the descending pathway. The ascending pathway carries sensory information from receptors to the brain via the spinal cord, and the descending pathway is composed of nerves that travel down the spinal cord to the rest of the body. The ascending pathway begins when a damaged cell releases cytokines which function as signaling proteins that modulate activity of the immune system. Cytokines then send signals to nearby nociceptors. If the stimulus is strong enough to reach the pain threshold of the nociceptor, an impulse is propagated along a sensory nerve fiber to the dorsal horn of the spinal cord where the signal will be relayed to a second order neuron via the release of a neurotransmitter called substance P which functions to increase nerve sensitivity to pain. The signal will continue to travel through the spinal cord and the brain stem until it reaches the thalamus. The thalamus is the relay station of the brain where the second order neuron synapses with a third order neuron so that the pain is perceived by the somatosensory cortex [6] (Figure 1). Since nociceptive pain is caused by damage to tissues, the sensation is typically described as sharp, aching, or throbbing. This is often experienced by individuals with cancer after surgeries such as mastectomies, thoracotomies, prostatectomies. In addition, treatments such as radiation therapy often cause damage to surrounding tissues, resulting in nociceptive pain [19].

### 5.2 Neuropathic Pain

Neuropathic pain is caused by damage to nerves responsible for relaying information between the brain and spinal cord and is usually experienced as a burning, stabbing, or tingling sensation with no apparent trigger from the environment. For this reason, it is also known as “pathologic pain” as it serves no apparent purpose in the body’s innate defense system. The mechanism of transmission of neuropathic pain is similar to that of nociceptive pain, but without the presence of a stimulus to activate a nociceptor [19]. In contrast, the damage is inflicted on a nerve further in the pain pathway causing it to spontaneously send impulses to the somatosensory cortex. Sodium ion channels (NaV) are responsible for regulating the excitability of neurons, and neuropathic pain results when the influx of sodium ions is high enough to stimulate a nerve impulse. For this reason, many pain blockers and local anesthetics block NaV channels to prevent nerve impulses that lead to the sensation of pain. Potassium channels (KV) are also involved in neuropathic pain as they are responsible for returning neurons to their resting state, decreasing neuropathic pain [20].

Neuropathic pain is extremely common in the cancer population due to side effects of various drugs. Chemotherapy administers cytotoxic drugs into the body, and radiotherapy emits high-energy beams such as x-rays and protons, and these treatments are usually combined to destroy cancer cells [21]. However, these methods are unable to distinguish between healthy cells and cancer cells, therefore neurons are often damaged, resulting in neuropathic pain. Drugs administered in chemotherapy also upregulate NaV downregulate KV which increases membrane excitability of neurons as this inhibits neurons from returning to its resting state. Tumors and inflammation are also common causes of neuropathic pain as this can compress and damage surrounding nerves [20].

### 5.3 Inflammatory Pain

Inflammation is an innate immune response produced by the body as a reaction to harmful stimuli. There are two types of inflammation: acute and chronic. Acute inflammation is initiated when pathogens or injured host cells enter tissues and encounter immune cells such as macrophages. Immune cells will then release chemicals called chemokines and this activates mast cells stimulating the release of a substance called histamine which will dilate nearby blood vessels in order to increase blood flow and permeability to the tissue. This causes symptoms of redness, swelling, and pain. Leukocytes will flow into the tissue in three phases. First, plasma fluid will release various antimicrobial mediators to destroy pathogens and stop bleeding. Then, neutrophils will squeeze through blood vessel walls and destroy bacteria. Neutrophils also release reactive oxygen species (ROS) to aid in killing pathogens [22]. Finally, monocytes arrive at the site where they differentiate into macrophages and remove remaining pathogens and damaged neutrophils via phagocytosis. Macrophages are recycled by the lymphatic system before returning to the bloodstream. Once this happens, immune cells begin producing anti-inflammatory mediators instead of pro-inflammatory cytokines. Chronic inflammation results when proinflammatory signals are constantly being produced at a given tissue site. Pro-inflammatory molecules will bind to nociceptors and activate NaV and the signal will be relayed toward the dorsal horn of the spinal cord to the somatosensory cortex where pain is sensed. Chronic inflammation will eventually lead to increased pain sensitivity due to the high levels of proinflammatory immune cells in tissues [20].

Chemokines that are responsible for recruiting leukocytes to affected tissues during the inflammatory response are also produced by malignant tumor cells and stimulate growth and division of cancer. This causes inflammation of affected tissues because leukocytes, such as neutrophils and macrophages, produce proinflammatory mediators such as TNF-α, interleukins, and interferons. In addition, chronic inflammation increases risk of cancer due to elevated levels of ROS which can damage DNA and result in mutations leading to malignancies [23].

## 6. Mechanisms of Action for Pain Relief

### 6.1 Beta-Endorphins

**Figure 2.**
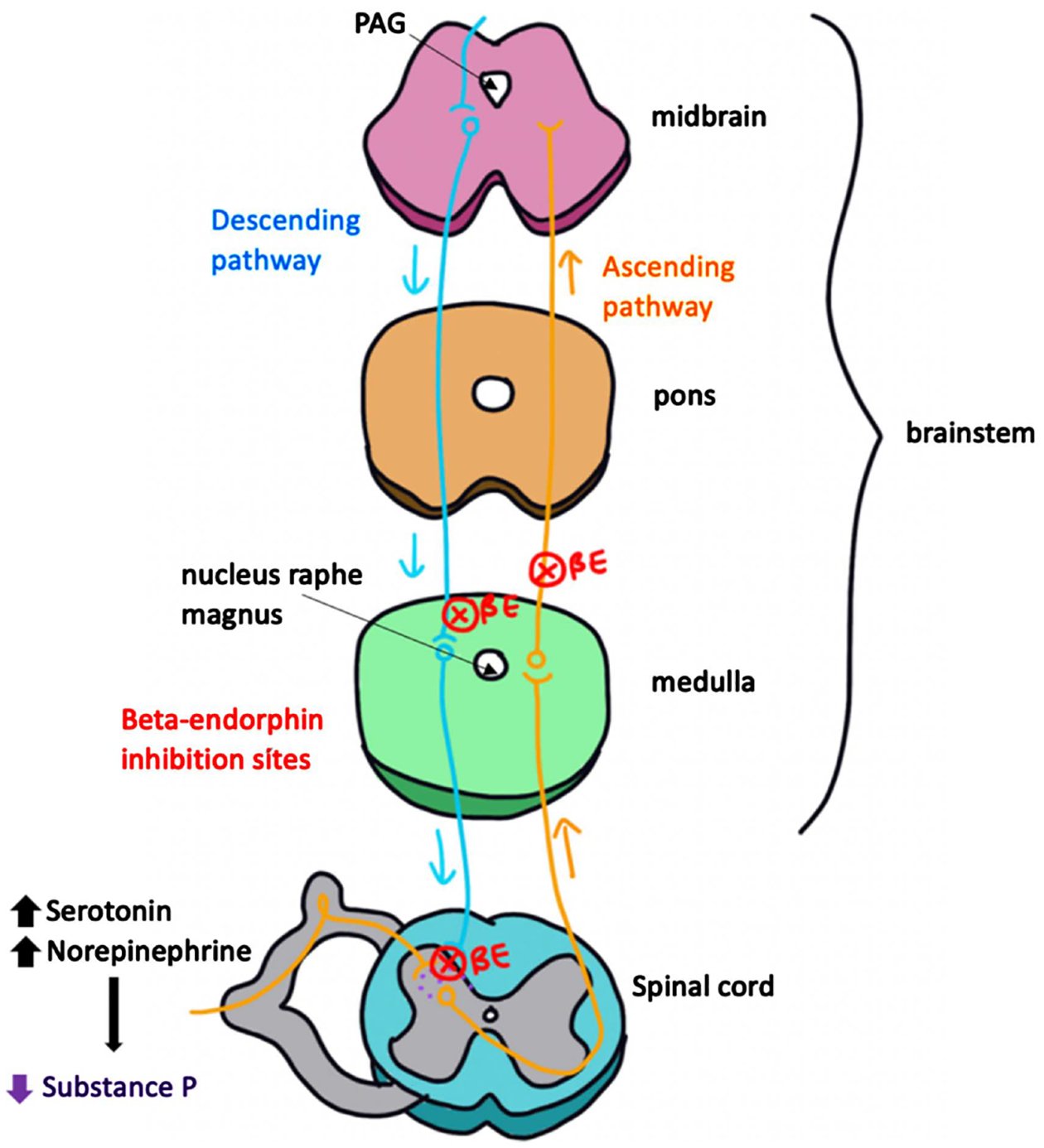
Beta-endorphins bind to neuronal sites throughout the ascending and descending pathway. The descending pathway neuron releases serotonin and norepinephrine at the dorsal horn, inhibiting the release of substance P by the 1st order neuron in the ascending pathway.

Beta-endorphins are a class of endogenous opioids found in the central and peripheral nervous system. They are neurotransmitters that are naturally produced by the body and bind to the same opioid receptors as pain-relieving drugs, such as oxycodone and morphine. Opioid receptors are located throughout the nervous system, and beta-endorphins can bind to receptors anywhere along the pain pathway to inhibit pain signaling to the somatosensory cortex. For example, a beta-endorphin can bind to a receptor on a second order neuron along the dorsal horn of the spinal cord, resulting in inhibition of the ascending pain pathway. It can also bind to receptors along the brainstem to activate the descending pathway of pain. In the descending pain pathway, a neuron in the area of the midbrain called the periaqueductal gray (PAG) synapses with a second neuron in a part of the medulla called the nucleus raphe magnus. The second order neuron travels down towards the dorsal horn of the spinal cord where it synapses with both the first and second order neurons in the ascending pathway and releases serotonin and norepinephrine to inhibit the release of substance P (Figure 2).

A 2020 randomized crossover study with 4 experimental trials was done with the goal of examining the effects of resistance training combined with low intensity blood flow restriction (BFR) and exercise induced hypoalgesia (EIH). 12 participants completed 4 separate trials performing a unilateral leg press exercise. The inclusion criteria consisted of recreationally active individuals ages 23 – 35 that were nonsmokers and had no comorbid cardiovascular, neurologic, musculoskeletal, or metabolic conditions. Participants refrained from strenuous exercise, alcohol, and caffeine 24 hours before each trial. A trial was completed at low-load resistance (30% 1RM), and this was repeated with low-pressure BFR.

Another trial was completed at high-load resistance (70% 1RM), and this was repeated with high-pressure BFR. Blood concentrations of beta-endorphins and PPTs were collected before exercise, 5 min after exercise, and 24 hrs after exercise. There was a significant increase (p < 0.01) in PPT and beta-endorphin levels in all trials at both 5 min and 24 hrs post exercise. The results of this study indicate that resistance training has both immediate and long-lasting effects on EIH which is mediated by the release of beta-endorphins. This is likely related to the stimulation of group III and IV muscle afferent fibers during exercise which synapse with neurons in the dorsal horn of the spinal cord. This muscle contraction stimulates the pituitary gland and peripheral nervous system to release beta-endorphins that bind to opioid receptors as an agonist in the descending pain pathway [24].

### 6.2 Anti-Inflammatory Cytokines

**Figure 3.**
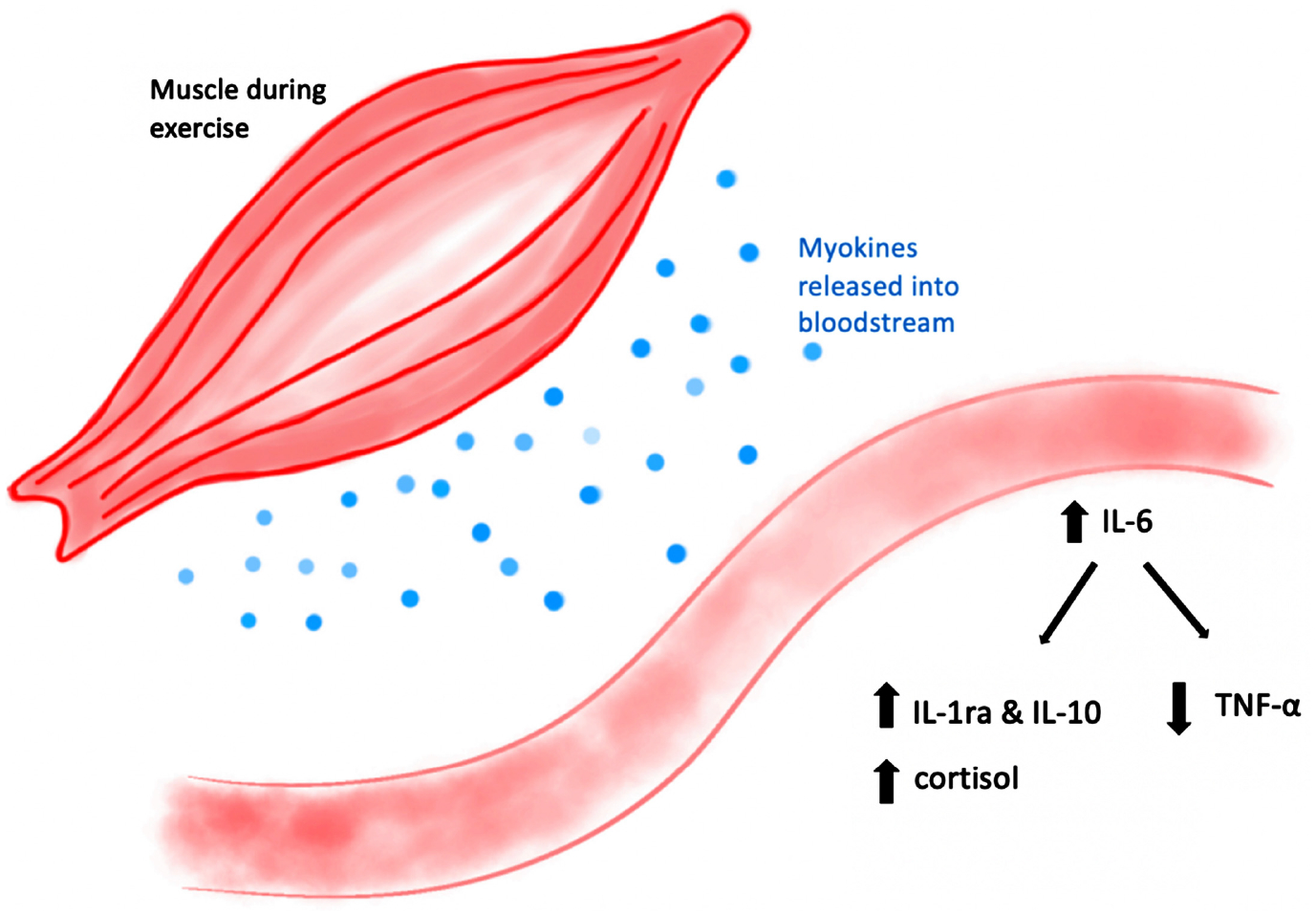
Muscles release myokines into the bloodstream during exercise. The anti-inflammatory myokine IL-6 upregulates the release of the anti-inflammatory cytokines IL-1RA and cortisol. IL-6 simultaneously inhibits the production of the inflammatory cytokine TNF-α.

During exercise, myokines (cytokines released from skeletal muscle during contraction) are produced when muscles contract to promote muscle hypertrophy. These myokines are involved in multiple anti-inflammatory processes. The anti-inflammatory myokine, Interleukin-6 (IL-6) has been shown to be present at higher concentrations post-exercise. IL-6 inhibits the pro-inflammatory cytokine, TNF-α, that is produced by macrophages and monocytes during the inflammatory response. IL-6 activates the production of other anti-inflammatory cytokines such as IL-1RA and IL-10 while simultaneously decreasing levels of TNF-α. IL-10 prevents synthesis of pro-inflammatory cytokines via inhibition of transcription or post-transcriptional mRNA degradation of corresponding genes. Secretion of IL-6 can be beneficial in reducing levels of chronic information. The anti-inflammatory hormone, cortisol, is also increased as a result of IL-6 production. Cortisol is an inhibitor of various pro-inflammatory cytokines including TNF-α. IL-1RA acts as an antagonist of the IL-1 proinflammatory receptor which inhibits intracellular signal transduction [4] (Figure 3).

With the goal of investigating the relationship between TNF-α and IL-6 during exercise, a 2003 experimental trial was conducted where 8 male participants received a bolus of TNF-α to induce low-grade inflammation. Prior to the study, physical exams were conducted on participants to ensure normal levels of white blood cells, C-reactive protein, blood glucose, insulin, coagulation proteins, and normal kidney and liver function. The participants were not taking any medications and had no history of illness within 2 weeks preceding the study. They were physically active with a maximal oxygen uptake between 4.0 – 5.0, and a weight range of 66 – 90.5 kg. Participants had a body mass index range of 21.3 – 26.5, and all were within 23 – 52 years in age. Each participant completed 3 experimental trials with 1 week between each trial. During the trials, the participants either rested for 3 hours, exercised for 3 hours, or were given an injection of IL-6 while they rested for 3 hours. The bolus was given 2.5 hours into the trial, and TNF-α levels were monitored for 2.5 hours after the bolus was given. Results showed significant differences between all three trials where lowest TNF-α levels were found in the trial with the IL-6 infusion and highest TNF-α levels were found in the trial at rest throughout the entirety of the 2.5 hours. This is evidence that exercise increases IL-6 levels, and that IL-6 inhibits inflammatory responses by decreasing levels of TNF-α. Furthermore, participants had significantly increased levels of epinephrine and norepinephrine in the blood in the exercise trial. These neurotransmitters also inhibit TNF-α, but elevated levels were not observed after the IL-6 injection, indicating that these neurotransmitters have anti-inflammatory mechanisms independent of IL-6. Because increased levels of the anti-inflammatory cytokine IL-6 were observed after each trial, it was concluded that exercise mediates mechanisms that provide relief to low-grade inflammation [25]. This is applicable to the cancer population as the metastasis of solid tumors is often associated with inflammatory cells such as cytokines and macrophages [26]. Implementing regular exercise will promote anti-inflammatory processes to relieve cancer pain and increase overall quality of life.

### 6.3 Endocannabinoids

**Figure 4.**
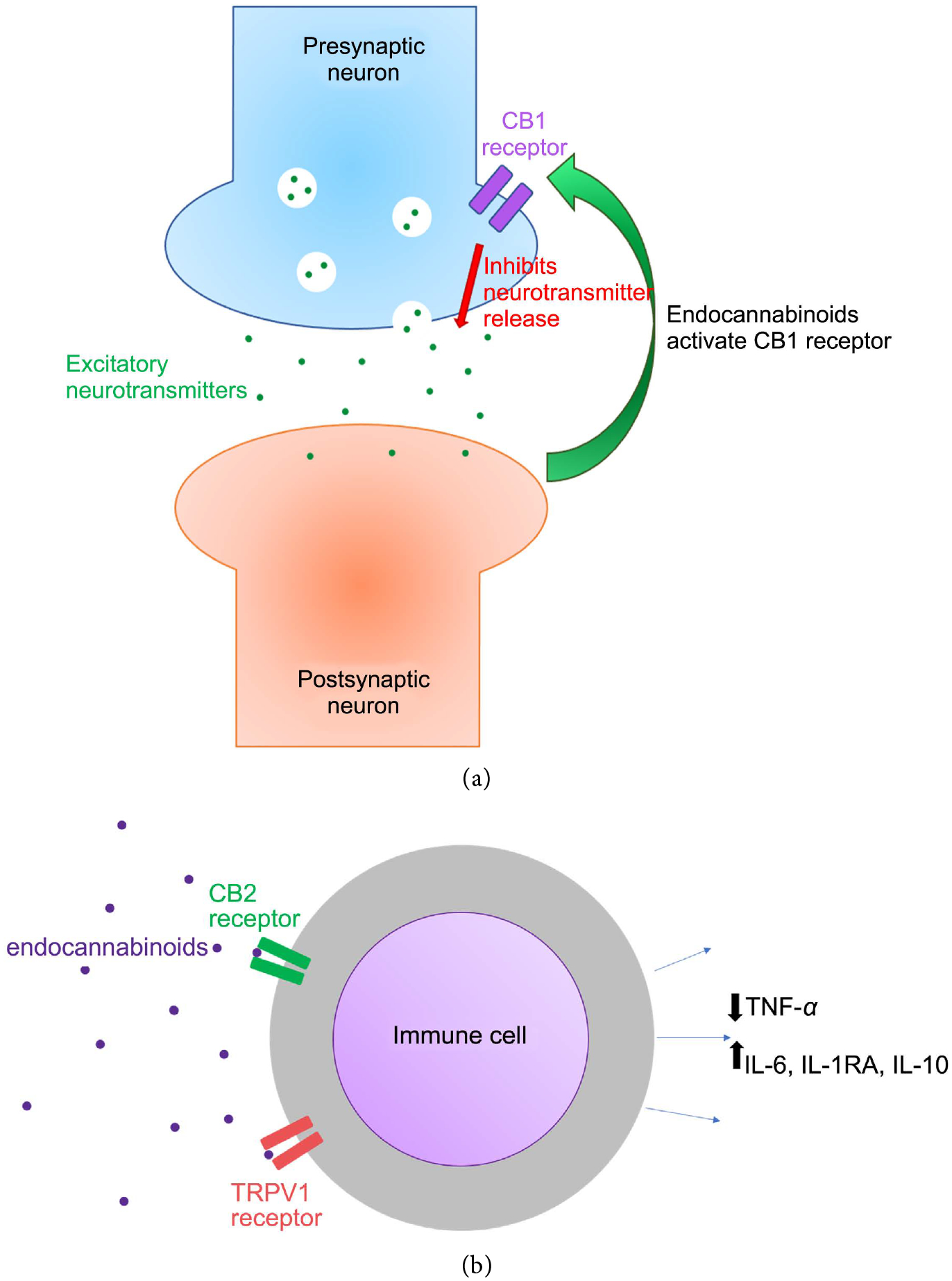
(a) Presynaptic neuron in the pain pathway releases excitatory neurotransmitters into the synaptic cleft activating the postsynaptic neuron. The postsynaptic neuron releases endocannabinoids which bind to CB1 receptors on the presynaptic neuron. This will act as a negative feedback loop to inhibit the release of excitatory neurotransmitters in the presynaptic neuron decreasing the sensation of pain. (b) Endocannabinoids bind to CB2 and TRPV1 receptors on immune cells inhibiting the release of the pro-inflammatory cytokine, TNF-α and activating the release of anti-inflammatory cytokines IL-6, IL-1RA, and IL-10.

Cannabinoids are substances found in cannabis or marijuana, and have been used to treat various conditions including cancer pain and inflammation. The chemical in cannabinoids responsible for pain relieving effects is identified as tetrahydrocannabinol (THC) binds to CB1 receptors in the brain and CB2 receptors in the peripheral tissues and immune cells. It was later discovered that the body naturally produces THC-containing compounds called endocannabinoids. The endocannabinoid system is an active pathway in pain perception and relief [27]. Similar to cannabinoids, endocannabinoids are lipid compounds that bind to CB1 and CB2 receptors. CB1 receptors are located on presynaptic neurons and help prevent excessive release of excitatory neurotransmitters. When neurotransmitters are released by a presynaptic neuron, and bind to the postsynaptic neuron, endocannabinoids are released by the postsynaptic neuron and move in a retrograde manner binding CB1 receptors on the presynaptic neuron. The presynaptic neuron is then stimulated to release GABA and glutamate to inhibit the release of neurotransmitters by increasing K^+^ conductivity to hyperpolarize the presynaptic neuron. CB1 receptors are highly expressed on areas of the brain involved in nociceptive pathways such as the PAG (primary control center for descending pain modulation) and the medulla (most inferior part of the brainstem connecting the brain to the spinal cord) (Figure 4(a)). When CB2 receptors are bound by endocannabinoids, immunomodulating pathways inhibit the production of inflammatory cytokines such as TNF-α and increase production of anti-inflammatory cytokines such as IL-10. Endocannabinoids also bind to anti-nociceptive TRPV1 ion channels, blocking the sensation of pain [8] (Figure 4(b)) (Table 3).

The endocannabinoid system is activated by exercise. In a 2012 study, 11 participants were assigned to exercise for 60 minutes of moderate exercise followed by 30 minutes of intense exercise followed by a 15-minute recovery. Blood was drawn before exercise, at the end of the 60 minutes of moderate exercise, at the end of the 30 minutes of intense exercise, and after the 15 minutes of recovery. Results indicated that plasma levels of the endocannabinoid anandamide, a lipid neurotransmitter that acts as a CB1 ligand, significantly increased during exercise and recovery (p < 0.001). From this, it was concluded that the endocannabinoid system is one of the mechanisms in which exercise leads to pain relief [28].

## 7. Exercise Increases Cancer Survivorship

Exercise is associated with significant improvements in both physiological and psychological functioning and overall quality of life in the cancer population. Regular exercise at a light intensity may reduce mortality rates in the breast cancer population. A 2005 study concluded that walking 3 – 5 hours per week significantly reduces risk of death. This is likely associated with lower levels of ovarian hormones in the bloodstream of women who perform regular exercise [29] [30]. These benefits were seen regardless of menopausal status. The ability of exercise to regulate female hormones can also reduce risk for breast cancer development as excess levels of estrogen, an ovarian hormone that regulates the female reproductive system, can bind to cell receptors that lead to uncontrolled proliferation of breast tissue.

Cancer treatments often result in decreased muscular strength and endurance, and implementation of regular resistance training is an effective method of counteracting muscular atrophy. Men with prostate cancer who exercise for 3+ hours per week had a 61% reduction in mortality (p < 0.03). This may be due to the ability of physical activity to decrease levels of excessive testosterone production, thus slowing tumor cell proliferation. In addition, physical fitness reduces activity of the sympathetic nervous system (SNS) when an individual is at rest. The SNS promotes tumor growth by increasing production of norepinephrine, a hormone that binds to beta-adrenergic receptors on tumor cells to stimulate cell growth. Furthermore, blocking beta-adrenergic receptors reduces risk of mortality in the prostate cancer population [31].

In the lung cancer population, exercise has been associated with a significantly decreased risk of mortality and recurrence. This is likely related to the ability of exercise to increase cardiorespiratory fitness, thus decreasing risk for hypertension and other cardiorespiratory comorbidities. Cancer-specific survival is significantly reduced in individuals with lung cancer who present with pre-existing cardiovascular conditions [32].

## 8. Application in Healthcare

The results of these studies indicate that resistance training is associated with various mechanisms of pain relief in multiple cancer populations. However, over 50% of medical school curriculums do not include courses that are focused on physical activity. Educating physicians and nurses on the benefits of exercise will allow exercise protocols to be more widely prescribed to patients as a method of symptom self-management. In addition, this could potentially decrease or eliminate the demand for other pain-relieving medications that may lead to other unpleasant symptoms and adverse health conditions.

The Theory of Symptom Self-management can be incorporated in follow-up studies in order to moderate the progression and intensity of exercise to avoid worsening other symptoms such as fatigue and to further improve pain-relieving effects of exercise. Although the standardized exercise programs utilized in these studies, allows for simplified and accurate analysis of data, the TSSM takes into account various factors that affect pain management such as age, sex, and comorbidities. The experimental design should evaluate physiological, psychological, and contextual characteristics of patients in order to develop a more holistic approach that is tailored to individual needs.

Follow up studies may require more complex data collection methods that include both qualitative and quantitative measures. For instance, conducting regular interviews with participants in addition to collecting blood work and using pain-rating scales so that exercise plans can be adjusted accordingly to avoid overtraining. This will allow outside factors and other symptoms to be taken into account in order to decrease pain while avoiding exacerbation of other symptoms to ultimately improve overall quality of life.

To confirm that pain-relieving effects of exercise in the cancer population can be sustained long-term, future studies may take measurements of inflammatory markers up to a year after the study is complete. Emphasis on PSE to encourage self-management behaviors will allow development of skills to self-manage pain. This is more likely to provide a long-term solution rather than temporarily masking symptoms (Table 4).

## 9. Conclusions

Pain is one of the most common symptoms in the cancer population, and it can be caused by a combination of factors including tumors, chemotherapy, surgery, and radiation therapy. In theory, resistance training is an effective method of decreasing nociceptive pain, neuropathic pain, and inflammation in the cancer population. The mechanisms in which resistance training leads to pain relief are similar to those of cancer pain medications such as morphine, oxycodone, and tramadol. As research continues, resistance training may be an effective way of relieving cancer pain and improving overall quality of life in individuals with cancer by reproducing pathways of pain relievers naturally.

Different types of pain involve various complex mechanisms of how pain is induced and perceived. Nociceptive pain is triggered by harmful stimuli on nerve endings, and signals are relayed to the brain via the spinal cord. Neuropathic pain is often the side effect of cancer treatment and occurs when nerves are chronically damaged. Inflammation is an immune response mediated by various pro-inflammatory cytokines that activate nociceptors and increase pain sensitivity. Regardless of the pain type, the results of the studies presented have indicated that resistance training is able to decrease pain through various mechanisms.

In the breast cancer population, resistance training is most effective in decreasing pain sensitivity when combined with aerobic exercise. Resistance training improves muscle strength which may increase PPT in those undergoing chemotherapy [3]. Combining resistance training and resistance training appears to be the most effective method to improve symptoms in the lung cancer population as well. A major concern in patients post-thoracotomy is significant decrease in cardiovascular endurance and strength [2]. Perioperative interventions involving both strength and aerobic training have been successful in maintaining cardiovascular function as well as managing pain [14]. In the prostate cancer population, ADT has been linked to various inflammatory disorders due to decrease in testosterone production. Full body resistance training with progressive overload is effective in reducing inflammation and preventing muscle loss during ADT. Implementing progressive resistance training increases the amount of testosterone secreted to maintain muscle mass and increases the amount of IL-6 released by muscles to decrease inflammation [7].

The studies in this review had limitations that prevent definite answers regarding the effectiveness of resistance training in cancer pain relief. Many studies had a small sample size, thus results cannot be generalized to the entire cancer population. In a majority of studies, additional treatments were imposed with resistance training and results may have been affected by a variety of factors. For example, compression of the exercising limb restricts blood flow. Furthermore, there is controversy over whether beta-endorphins are effective in pain relief as they are unable to cross the blood brain barrier [24]. Overall, resistance training has shown promising effects in pain relief, but more studies need to be conducted to determine its degree of effectiveness and its exact pain-relieving mechanisms.

Exercise has been associated with increased survivorship in the cancer population [30] [31] [32]. This is due to regulation of hormones such as estrogen, progesterone, epinephrine, and norepinephrine in addition to regulation of the sympathetic nervous system. Consistent physical activity decreases tumor proliferation and lowers risk of comorbidities, lowering the risk of mortality in the breast cancer, lung cancer, and prostate cancer populations.

The Theory of Symptom Self-management provided the framework for this review. Stronger evidence of pain relief was indicated in the studies that adjusted the intervention based on the abilities of the individual participants (e.g. progressive overload). In addition, the studies that evaluated a wide variety of patient characteristics and symptoms in addition to pain levels (e.g. HRQoL, cardiorespiratory functioning, body mass) had more significant improvements in pain-relief, indicating that interrelationships between symptoms should be evaluated for optimal results.

## Figures and Tables

**Table 1.** Inclusion and exclusion criteria used in PubMed and Google Scholar literature searches.

criteria	inclusion	exclusion
population	Individuals with early-stage breast, lung, or prostate cancer	Terminal cases or individuals undergoing treatment without curative intent. Other types of cancer
design	Randomized controlled trial	non-randomized/non-controlled interventions
treatment	Exercise in the form of resistance training or aerobic training	
outcomes	Pain as a primary or secondary outcome (can be measured in multiple ways *i.e*.rating scale, PPT, inflammatory markers)	
Publication type	Peer-reviewed articles	Any article not subject to peer review

**Table 2.** Summary of each exercise study. Contains article citation, population studied, intervention strategy, treatment groups, outcomes measured, timing of intervention, and study design.

Article	population	intervention	groups	Outcomes measured	Timing	Study design
Mijwel, Backman, Bolam 2018 *Breast Cancer Research and Treatment*	260 women with stage I-III breast cancer 18 – 17 years undergoing chemotherapy for curative intent	Aerobic training, HIIT, resistance training 2x/week for 16 weeks	AT-HIIT, RT-HIIT	Cardiorespiratory function, muscle strength, body mass, pressure pain threshold	Undergoing chemotherapy	Randomized controlled trial
Omar M, Gwada, Omar G 2019 *Journal of Cancer Education*	60 women ages 18+ years with breast cancer related lymphedema symptoms	Low-intensity resistance exercises and compression garment 3x/week for 8 weeks	Low-intensity resistance training alone, Low-intensity resistance training with resistance garment	lymphedema volume determined by percentage reduction of excess limb volume	N/A	Randomized controlled trial
Fugazzaro, Costi, Manini, 2017, *BMC Cancer*	40 patients with stage I-II NSCLC	Aerobic and resistance training 2 – 3x/week for 6 months	Intervention group participated in aerobic and resistance training	Long-term exercise capacity measured by 6-min walk test, pain relief	Begins before surgery; results measured at 1 month and 6 months post-surgery	Randomized controlled trial
Brocki, Andreasen, Nielsen, 2013, *Lung Cancer*	78 early-stage lung cancer patients	Aerobic exercises, resistance training, dyspnea management 1 hr/week for 10 weeks	Intervention group participated in aerobic and resistance training	HRQoL (survey), 6-min walk test, lung function (spirometry)	Treatments imposed 3 weeks to 4 months after surgery. Results measured immediately after surgery, and at 4 & 12 months. Thoracotomy was performed in 77% of participants	Randomized controlled trial
Nilsen, Raastad, Skovlund 2015 *Acta Oncologica*	58 males with prostate ca undergoing ADT	High-load strength training 3x/week for 16 weeks	Intervention group participated in heavy resistance training	Lean body mass, bone density (DXA), physical strength (1RM), HRQoL (questionnaire)	Undergoing ADT	Randomized controlled trial

**Table 3.** Biochemicals studied and their respective functions.

Biochemical	Function
cytokines	Broad category of signaling proteins that regulate and recruit immune cells
Substance P	Neurotransmitter that increases pain sensitivity
chemokines	Subcategory of chemokines that recruit leukocytes during the inflammatory response
histamine	Released by mast cells of immune system to dilate blood vessels, leading to swelling and inflammation
TNF-*α*	Inflammatory marker produced by immune cells
Beta-endorphins	Class of endogenous opioids naturally produced by the body that bind to pain-relieving receptors in the nervous system
Interleukin-6 (IL-6)	Anti-inflammatory myokine
endocannabinoids	Lipid compounds synthesized naturally by the body that mediate pain perception and relief.
THC	Active chemical in cannabinoids that bind to CB1 and CB2 receptors leading to pain relief

**Table 4.** Abbreviations and their respective definitions.

Abbreviation	Definition
TSSM	Theory of Symptom Self-management
PPT	Pressure pain threshold
IL-(6/1RA/10)	Interleukin (6/1RA/10)
PSE	Perceived self-efficacy
SNS	Sympathetic nervous system
THC	tetrahydrocannabinol
NSCLC	non-small cell lung cancer
SCLC	small cell lung cancer
HIIT	high intensity interval training
RT	Resistance training
AT	Aerobic training
ROM	Range of motion
IG	Intervention group
CG	Control group
RPE	rating of perceived exertion
SF36	short-form health survey questionnaire
ADT	Androgen deprivation therapy
RA	rheumatoid arthritis
LBM	Lean body mass
DXA	dual x-ray absorptiometry
NaV	Sodium channel
KV	Potassium channel
ROS	Reactive oxygen species
PAG	periaqueductal gray
BFR	Blood flow restriction
EIH	exercise induced hypoalgesia
